# Stress and the dopaminergic reward system

**DOI:** 10.1038/s12276-020-00532-4

**Published:** 2020-12-01

**Authors:** Ja-Hyun Baik

**Affiliations:** Molecular Neurobiology Laboratory, Department of Life Sciences, Korea University, Seoul, 02841 South Korea

**Keywords:** Molecular neuroscience, Motivation

## Abstract

Dopamine regulates reward-related behavior through the mesolimbic dopaminergic pathway. Stress affects dopamine levels and dopaminergic neuronal activity in the mesolimbic dopamine system. Changes in mesolimbic dopaminergic neurotransmission are important for coping with stress, as they allow adaption to behavioral responses to various environmental stimuli. Upon stress exposure, modulation of the dopaminergic reward system is necessary for monitoring and selecting the optimal process for coping with stressful situations. Aversive stressful events may negatively regulate the dopaminergic reward system, perturbing reward sensitivity, which is closely associated with chronic stress-induced depression. The mesolimbic dopamine system is excited not only by reward but also by aversive stressful stimuli, which adds further intriguing complexity to the relationship between stress and the reward system. This review focuses on lines of evidence related to how stress, especially chronic stress, affects the mesolimbic dopamine system, and discusses the role of the dopaminergic reward system in chronic stress-induced depression.

## Neuroscience: finding reward in stressful situations

The brain’s response to stressful conditions is linked to mechanisms for processing rewarding stimuli, but the nature of this connection remains poorly understood. Studies indicate that how animals cope with acute or chronic stress relies in part on dopamine, a neurotransmitter that produces ‘reward’ signals in the brain. Ja-Hyun Baik, Korea University, Seoul, South Korea, reviews how stress influences dopamine-mediated signaling, in particular exploring the hypothesis that the nature of stressful stimuli specifically determines the behavior induced by dopamine and the way signals are perceived in terms of stress or reward. However, the author also notes the many unanswered questions around the neurological mechanisms underlying chronic stress, and the depression and lack of pleasure produced by stress. Further research is needed to understand the role of dopamine signaling.

## Introduction

Hans Selye, who coined the term “stress” to describe ‘the non-specific response of the body to any demand for change’ in 1936, stated in his later years that ‘Everyone knows what stress is, but nobody really knows about stress’^1,2^. A tremendous number of studies have established that stress is a crucial factor in psychopathology, particularly the development of depression. Numerous animal studies have shown that stressful events can induce despair and altered responses to reward, which are characteristic symptoms of depression in humans^3,4^. Acute stress appears to increase reward sensitivity to allow successful coping with the recruitment of appropriate reward-related neural connections. However, chronic stress results in blunted reward sensitivity, which can induce the loss of pleasure or a lack of motivation, that is, anhedonia, which is one of the core features of depression^3–6^.

The mesolimbic dopaminergic system is known as a major reward-related center in the brain. Changes in dopaminergic neurotransmission can modify and alter behavioral responses to different environmental stimuli that are associated with reward anticipation. Stressful events often include aversion and avoidance, which may negatively regulate the dopaminergic reward system. Evidence from human and animal studies suggests that modulation of the dopaminergic reward system is necessary for monitoring and selecting the optimal process for coping with these aversive events, indicating that dopaminergic regulation plays an important role in the pathophysiology of stress-related behaviors^7–10^.

Recent studies using cell- and circuit-level labeling and manipulation techniques have provided novel insights into the neurobiology of stress in association with the reward system. In this review, I will review and discuss current findings on the effects of stress, especially chronic stress, on the mesolimbic dopaminergic system and on the role of the dopaminergic reward system in the stress response.

## Dopamine reward pathway

**Fig. 1:**
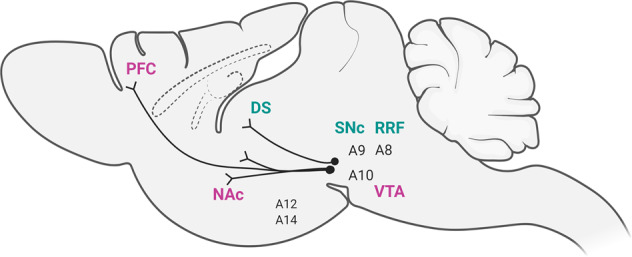
Dopaminergic pathways in the brain. Schematic illustration of dopaminergic pathways in the central nervous system in the rodent brain. A8 cells are predominantly found in the retrorubral field (RRF), and A9 neurons are located in the substantia nigra pars compacta (SNc) and project to the dorsal striatum (DS), constituting the nigrostriatal pathway. The mesolimbic and mesocortical pathways include projections from A10 cells in the VTA to the nucleus accumbens (NAc), prefrontal cortex (PFC), and other limbic areas (which are not shown here). Other distinct groups of cells constituting the tuberoinfundibular pathway, namely, A12 cells and A14 cells in the hypothalamus, are shown.

Dopamine (DA), as a predominant catecholamine, is produced in the substantia nigra (SN) and the ventral tegmental area (VTA) in the midbrain. DAergic neurons from the SN and VTA project to numerous different areas of the brain. DAergic neurons can be identified by immunohistochemistry for tyrosine hydroxylase (TH), the rate-limiting enzyme of DAergic synthesis. These DA-producing cell groups are designated group A cells, a class of cells containing catecholamines, primarily noradrenaline and DA, that can be subdivided into the DA-containing cell groups A8 through A16. A8 cells are predominantly found in the retrorubral field (RRF), and A9 neurons are located in the substantia nigra pars compacta (SNc) (Fig. 1) and project to the dorsal striatum (DS), constituting the nigrostriatal pathway. A8 neurons are generally considered an extension of the A9 cell group, and these cells contain cells that project to striatal, limbic, and cortical areas^11–15^. The nigrostriatal pathway is involved primarily in the control of motor function but also in goal-directed behaviors, including reward-related cognition and learning. A10 cells are located in the VTA, and from the VTA, A10 cells project to the nucleus accumbens (NAc), prefrontal cortex (PFC), and other limbic areas. This group of cells constitute the mesolimbic and mesocortical pathways (Fig. 1) and is known to be involved in reward-related positive and negative reinforcement, incentive salience, aversion-related cognition, and decision-making^16^.

In rodents, analysis of the total number of TH-immunoreactive neurons in the midbrain revealed approximately 45,000 TH-positive neurons in rats and approximately 21,000–30,000 TH-positive neurons in mice^12–14^. It is estimated that there are 160,000–320,000 TH-immunoreactive neurons in the primate midbrain and approximately 400,000–600,000 TH-positive neurons in the human midbrain^12–14^. It has been reported that A8 cells account for approximately 5%, and A9 and A10 cells account for approximately 95% of these neurons, with an almost equal distribution in rodents, while the A9 cell population constitutes the majority of cells in the midbrain in primates and humans (approximately 70%), indicating a considerable expansion of the SN in primates and humans compared to rodents^12–14^. Another distinct group of cells constitutes the tuberoinfundibular pathway. These cells are distributed throughout the arcuate nucleus (A12 cells) and periventricular nucleus (A14 cells) of the hypothalamus, project to the pituitary, and are involved in regulating the release and synthesis of pituitary hormones, primarily prolactin^15^ (Fig. 1).

Dysfunction of the DAergic system is the hallmark of the pathology of a number of neuropsychiatric disorders, such as Parkinson’s disease, drug addiction, depression, and schizophrenia. In the following section, we will focus on the VTA and NAc as the core components of the reward system, which is closely associated with stress-related behaviors.

### Ventral tegmental area

**Fig. 2:**
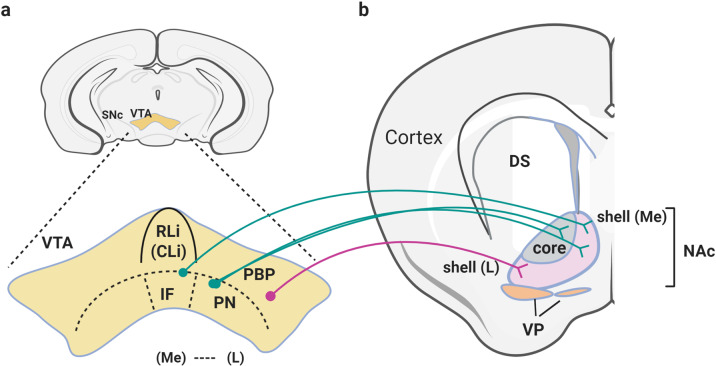
Ventral tegmental area (VTA) and nucleus accumbens (NAc). **a** Subareas within the VTA, in the lateral part of the VTA, the parabrachial pigmented area (PBP), which is continuous with the rostral SN (substantia nigra), and the paranigral nucleus (PN), which is rather restricted to the caudal VTA, are shown, and these regions are rich in DA neurons. The interfacial nucleus (IF) is found in the medial part of the VTA, the rostral linear nucleus of the raphe (RLi) is located in the rostral part of the VTA, and the caudal linear nucleus (CLi) is found in the caudal part of the VTA. The medial (Me) and lateral (L) parts of the VTA are indicated. **b** Schematic illustration of the NAc. The core and the shell (the medial and lateral parts of the shell are represented by (Me) and (L), respectively) of the NAc are showed together with the ventral pallidum (VP). DAergic neurons in the medial VTA (IF, PN, and medial PBP), which are schematically represented here by green projections, selectively project to the medial shell and core of the NAc, while DAergic neurons in the lateral VTA (lateral PBP), represented as pink projections, project to the lateral (L) shell of the NAc.

The VTA consists of DAergic neurons (TH-positive, approximately 60–65% of neurons in the VTA), ‘GABA (γ‐aminobutyric acid)ergic’ (35%) and a relatively small portion of glutamatergic neurons (2–3% of neurons in the VTA)^17–19^. VTA DAergic neurons can be subdivided according to their relative location along the rostral/caudal or medial/lateral axes. Within the lateral part of the VTA, the parabrachial pigmented area (PBP), which is continuous with the rostral SN, and the paranigral nucleus (PN), which is rather restricted to the caudal VTA, extend to the ventromedial part of the VTA, and these regions are rich in DAergic neurons^17–19^ (Fig. 2a). The interfacial nucleus (IF) is located in the medial part of the VTA, the rostral linear nucleus of the raphe (RLi) is found in the rostral part of the VTA and the caudal linear nucleus (CLi) is located in the caudal part of the VTA^17–19^ (Fig. 2a). Neurotransmitters, receptors, transporters, and neuropeptides are differentially expressed within the subregions of the VTA, highlighting the heterogeneity of the VTA.

To identify DAergic cells within the VTA for electrophysiological studies, several key features of midbrain DAergic neurons can be used: 1) a slow (2–10 Hz) firing rate, which may include burst firing; 2) hyperpolarization-activated inward currents (*I*_h_) generated by hyperpolarization-activated cyclic nucleotide-regulated cation channels (HCN channels);^19–21^ and 3) dopamine D2 receptor (D2R) agonist-induced hyperpolarization^19–21^. However, there is still some variability in non-DAergic neurons, as large *I*_h_ can be observed in non-DAergic neurons within the VTA^19^. The correlation between *I*_h_ and DAergic neuron phenotype is often considered an important criterion and can be applicable for conventional DAergic neurons in the dorsolateral region of the VTA, specifically the anterior PBP^19–21^. However, other subregions in the medial VTA, such as the medial PN and medioventral part of the PBP, contain nonconventional DAergic neurons with a distinct electrophysiological profile, displaying no or very little *I*_h_ with different action potential firing patterns^19–22^.

In addition, these conventional/nonconventional DAergic neurons send different projections to the NAc. DAergic neurons in the dorsolateral region of the VTA (lateral PBP) project to the lateral shell of the NAc, while DAergic neurons in the ventral medial VTA (PN and medial PBP) project to the medial shell and core of the NAc and medial prefrontal cortex (mPFC). DAergic neurons in the dorsomedial VTA, CLi and IF project to the medial shell of the NAc^21,23^. Another complexity involves the differential expression of neurotransmitter transporters or receptors. For example, DAergic neurons show differential expression of molecules such as vesicular monoamine transporter 2 (VMAT2) and dopamine transporter (DAT) as well as vesicular glutamate transporter (VGluT2) and dopamine D2 receptor (D2R)^17,24^. Within the lateral part of the VTA (which includes the lateral PBP and lateral PN), DAergic neurons express VMAT2 and DAT or D2R, but in the medial VTA (which includes the medial PBP, medial PN, IF, and RLi), DAergic neurons express VGluT2 but not VMAT2, DAT, or D2R^17,24^. Therefore, the difference in anatomical wiring and molecular features as well as the heterogeneity in the electrophysiological characterization of DAergic neurons within the VTA can contribute to the different functions of subpopulations of VTA neurons.

### Nucleus Accumbens

The NAc, which is the ventral area of the striatum and is distinct from the dorsal striatum, which includes the caudate and putamen, is recognized as the main center for reward-related behavior, including learning and motivational processes, and receives dopaminergic inputs from the VTA. The NAc has two subregions, a central core that is medially and ventrally located surrounding the anterior commissure and an outer shell that is located lateral, extends around the core, which can be further subdivided into lateral and medial shell regions (Fig. 2b). However, it should be noted that this division between the core and shell is applicable to the caudal part of the accumbens, whereas the rostral part is referred to as the rostral pole of the accumbens^25,26^. The core and the shell of the NAc differ in their histochemical, electrophysiological, and molecular and cellular characteristics and make different afferent and efferent connections, suggesting that there are differences in their functions as well^26–29^. One of the molecular markers that can be used to differentiate the shell from the core in the NAc in rats as well as primates and humans is the calcium-binding protein calbindin-D_28K_^30,31^. The core exhibits strong calbindin-D_28K_ immunoreactivity, while the shell shows weak to no immunoreactivity for this protein. Additionally, it has been reported that the expression of substance P, enkephalin and calretinin is different in the core and shell^26,30,32^.

In addition to the dopaminergic inputs from the midbrain, including the VTA and the medial part of the SNc, the NAc receives glutamatergic inputs from the hippocampus, PFC (prelimbic cortex and infralimbic cortex), basolateral amygdala, and thalamus^31,33–35^. As mentioned earlier, DAergic neurons in the medial posterior VTA (PN and medial PBP) selectively project to the NAc medial shell and core, while DAergic neurons in the lateral posterior and anterior VTA (lateral PBP) project to the NAc lateral shell^21,23^. The core receives inputs from the dorsal part of the mPFC, whereas the shell receives cortical inputs from the more ventral part of the mPFC^31,34,36^. The core receives inputs from the anterior part of the basolateral amygdala, and the shell receives inputs from the posterior parts of the basolateral amygdaloid nucleus^37^.

Regarding outputs, the NAc core and shell send distinct efferent projections. The dominant outputs of the NAc project to areas including the ventral pallidum (VP), the medial part of the globus pallidus (GP), and the SN as well as the lateral hypothalamus (LH)^31,38,39^. The outputs of the core send similar projections as the dorsal striatum, include projections to the dorsolateral part of the VP, the medial part of the internal segment of the GP, and direct projections to the dorsomedial part of the substantia nigra pars reticulata (SNr)^27,39–41^. The VP projects to the dorsomedial part of the subthalamic nucleus but also to the SN, thus participating in basal ganglia circuits^31,41,42^. The medial parts of the internal GP and the SN project to the thalamic nuclei, which are reciprocally connected with prefrontal areas that, in turn, project to the core of the NAc. Thus, it is likely that the NAc core sends projections predominantly to areas of the motor system involved in execution of action. The NAc shell projects preferentially to the medial VP, LH, VTA, SNr, bed nucleus stria terminalis, and central amygdala^39,40^. The shell is thus predominantly connected to the subcortical limbic system. Based on this anatomical segregation and connection, it has been suggested that the core and shell have distinct functions, particularly regarding reward-related behaviors. For instance, the core is through to be involved in learning and action selection during goal-directed behavior, whereas the shell appears to be involved in more emotional/motivational value-related responses^43–45^.

**Fig. 3:**
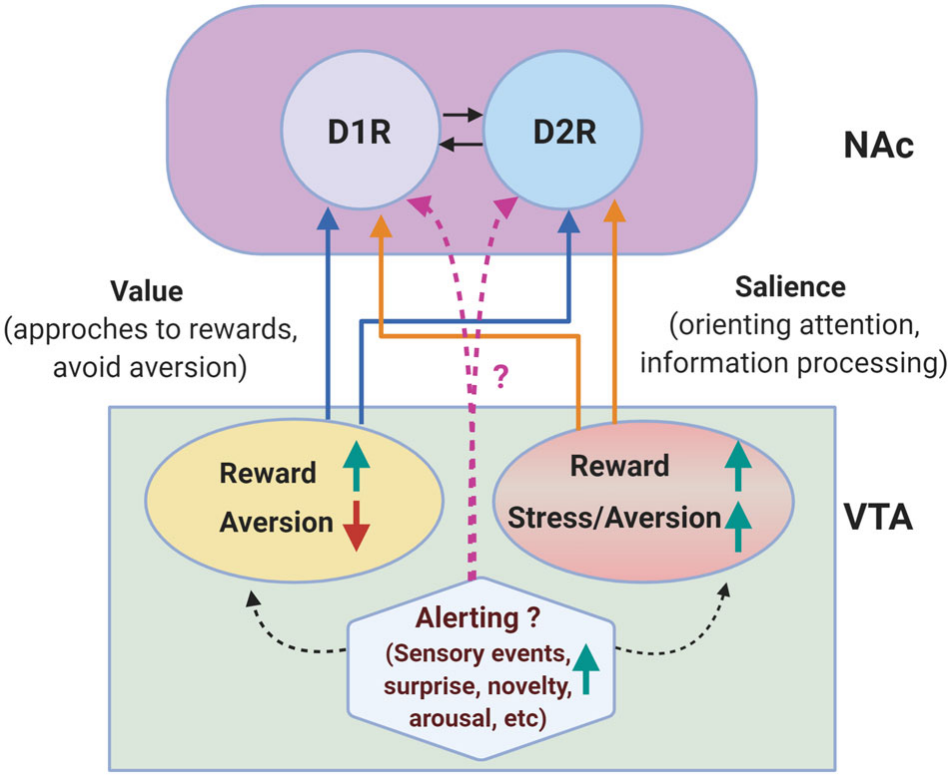
Reward and stress/aversion signals in the VTA-NAc pathway. Based on recent findings, a diverse population of DAergic neurons that are excited (upward green arrow) by reward and inhibited (downward red arrow) by aversive stimuli and another population excited by both reward and aversive events are present in the VTA^16,108,109^. In addition to being regulated by reward and aversive stimuli, DAergic neurons can be excited by numerous alerting signals (sensory events, surprise, novelty, arousal, attention, and salience), which do not necessarily associated have reward value^110^. These value, salience, and alerting signals can cooperate to coordinate and control motivated behavior^16^ and may ultimately be important not only for reward value but also for supporting specific forms of adaptive behavior to react and cope with changes in the environment. These different DAergic neuronal populations may activate DAergic receptors in the NAc, but this process remains to be elucidated; here, the core and the shell of the NAc were not depicted. Two types of GABAergic medium spiny neuron (MSN) populations (90–95%) that constitute the majority of NAc neurons and express either dopamine D1R (D1-MSNs) or D2R (D2-MSNs) are represented as D1R and D2R, and their mutual connections are depicted by arrows. Other limbic areas are not described in the present figure.

In addition to being divided into the core and shell, the NAc, like the dorsal striatum, is largely composed of two types of GABAergic medium spiny neuron (MSN) populations (90–95%) that express either dopamine D1R (D1-MSNs) or D2R (D2-MSNs) (Fig. 3). The efferents of D1-MSNs project to the midbrain, VTA, SN, and VP, whereas those of D2-MSNs project to the VP and subthalamic nucleus before reaching the VTA^27,46,47^. D1-MSNs are relatively homogeneously distributed throughout the core and shell of the NAc, while D2-MSNs are heterogeneously distributed in the ventral and caudomedial parts of the NAc shell^48^. Interestingly, approximately 17% of MSNs in the shell, which is a much greater percentage than in the dorsal striatum and the core (5–6%), coexpress D1R and D2R at detectable levels^49^, however, it is not yet known how these cells translate D1R/D2R signals in response to DA stimulation.

## How stress affects the VTA-NAc dopaminergic system

### Stress-induced changes in the DA level

Initial evidence for the stress-induced regulation of the DAergic system demonstrated that DA release or DA metabolism, particularly in the mesolimbic DAergic system, is changed in response to stressful stimuli. DA release can be enhanced or inhibited on the basis of the intensity, duration, and avoidability of the stressor. It has been reported that the curve of DA release in response to stress exposure is an inverted U shape, with mild to moderate stressors that are novel, short-lasting, or controllable having activating effects on DA release^50,51^. In contrast, intense, chronic and unpredictable stressors have inhibitory effects on DA release induced by prolonged uncontrollable and unavoidable stress^50,51^. As reviewed in detail in a review by Holly and Miczek^19^, rodents exposed to acute restraint and immobilization stress (for 10–240 min) showed an immediate ~125–150% increase in the DA level by in the NAc and a larger range of increase (~139–250%) in the mPFC. Acute foot shock stress (10–30 min) also induced a ~135–230% increase in extracellular DA levels in the NAc and a ~150–250% increase in the mPFC^19^. Other stressors, such as tail pinch, short-term handling, and psychological stress, such as social threat or predator odor exposure, also induced a similar increase in DA levels in the NAc and the mPFC^19^. These data indicate that acute stress generally increases extracellular DA levels in the mesolimbic mesocortical DA system and that only minimal increases are observed in the striatum^7,8,52–55^.

Acute or short-term stress induces a change in the DA level or midbrain DAergic neuronal activity, and such a change in DAergic neuronal activity appears to promote reward-related neural connectivity by, for example, enhancing learning of cue-reward associations^56^; such acute events do not induce depressive behavior, while chronic repeated stress usually results in depressive behavior. As reported and validated by a number of studies, exposure of animals to chronic stress results in depressive-like behaviors. Current well-accepted chronic stress paradigms include chronic restraint stress, chronic social defeat stress, and chronic unpredictable mild stress (CUMS).

Chronic restraint stress is induced by placing animals in a restrainer (ventilated and transparent) for 2–6 h per day for 10–28 days. After chronic restraint stress, rodents display depressive-like behavior, as validated by different panels of behavioral tests, such as the sucrose preference test, forced swim test, and tail suspension test^57–59^.

The chronic social defeat stress (CSDS) protocol involves the introduction of a single male (intruder) to the home cage of a larger resident male mouse (aggressor, resident) for 5–10 min, during which the intruder is defeated by the resident mouse^60,61^. After this physical interaction, the residents and intruders are maintained in sensory contact for 24 hr. Each day, the experimental intruder mice are exposed to the home cage of a new resident, and this procedure is repeated for 10 consecutive days^60,61^. After CSDS exposure, social interaction time is measured, and two phenotypes can be distinguished: susceptible mice, which show depressive behaviors (a reduction in social interaction), and resilient mice, which do not exhibit depressive behaviors^60,61^.

The CUMS or chronic mild stress (CMS) procedure involves exposing rodents to a series of mild (that are in reality not that mild), unpredictable stressors (overnight illumination, cage tilt, damp bedding, unpleasant noises, home cage changes, food/water deprivation, etc.) in a random order for several weeks (2–12 weeks). This stress protocol induces persistent depressive behaviors and seems to mimic the stress-induced depression observed in depressed patients^58,62^.

There have been contradictory findings related to the effects of chronic stress on changes in DA release, likely due to differences in the stress protocol used and technical issues related to measuring the DA level. Variable results have been reported by studies that used the chronic restraint stress protocol. No change in the DA level was found after 10 min or 2 h of restraint stress per day for 10 consecutive days^63,64^, as measured by high-performance liquid chromatography (HPLC). Repeated restraint stress for 1 hr/day for 6 days resulted in a progressive reduction in stress-induced DA release, as measured by in vivo microdialysis^65^. In contrast, measurement of DA levels in the NAc of rats restrained for 15 min/day for 5 days by high-speed chronoamperometry showed that the effect of restraint stress-induced DA release was significantly greater on Day 5 than on the other days^66^.

It has been reported that CUMS for 2 weeks or 30 days does not affect the DA level in the NAc, as measured by microdialysis^67,68^. However, Wilner et al. and Stamford et al. reported that 3–7 weeks of exposure to CUMS increases the DA level in the NAc but not in the dorsal striatum, as determined by fast cyclic voltammetry and HPLC, respectively^69,70^. Prolonged exposure to unavoidable stressors results in decreases in DA and DA metabolite levels in the NAc of stressed animals. Rats exposed to unavoidable stress for 3 weeks in the electric shock paired escape test show reduced DA release in the NAc shell^71^. On the other hand, chronic food restriction, which can be considered an intense stressor, leads to a reduction in body weight to 70–80% of the normal level and a marked decrease in basal extracellular DA levels in the NAc of up to 50%, as measured by in vivo microdialysis^72^. No such change was observed in the dorsal striatum or mPFC, indicating that the VTA-NAC pathway might be involved in this chronic food restriction-induced stress^72^.

### Effect of chronic stress on VTA DAergic neuronal activity

Considerable evidence suggests that the excitability of mesolimbic dopaminergic neurons in vivo can mediate an individual’s responses to chronic stress, but intriguingly, the regulation of VTA neuronal activity showed different patterns depending on different stress paradigms.

DAergic neurons can exhibit two different patterns of DA release, namely, tonic and phasic, based on their firing properties^73^. It has been suggested that low-frequency, irregular, single-spike tonic firing of DAergic neurons generates a low basal level of extracellular DA^73^, while burst firing, or phasic activity, is associated with reward-related cues and is believed to be the functionally relevant signal for the postsynaptic modulation mediating reward-directed and goal-directed behavior^73–76^.

It has been reported that firing rate and burst firing was increased in the VTA DA neurons in rat after a single exposure to restraint stress of 30 min^77^. Similarly, Valenti et al., reported that acute restraint stress (2 hr) increase in VTA DA neuron population activity, which represents the number of DA neurons firing spontaneously, with a significant increase in the average percentage burst firing but without affecting the average firing rate^78^.

Animals exposed to the chronic restraint stress for 10 days displayed a significant increase in DA neuron population activity within the VTA but no change in the average firing rate or the average percentage of burst firing^78^. It has been suggested that population activity and burst firing are associated but regulated differently by distinct afferent systems^78,79^. Increase in DA neuron population activity has been proposed to modulate tonic extrasynaptic dopamine levels and this population activity provides to set VTA neurons ready for ‘responsive state’ to phasic events^78,79^. In other words, only neurons that are in tonic firing state can be phasically activated by the relevant salient stimulus (either threatening or rewarding)^73,78,79^. Most likely, this hypothesis can explain why both reward-related events and stressful events increase VTA DAergic neuronal activity; this increase in activity may establish a responsive state, allowing the regulation of reactivity to changes in the environment^73,78,79^.

As mentioned earlier, animals exposed to CSDS can be divided into two groups, a susceptible (depressed) group and a group that is resilient to stress. Interestingly, following exposure to this chronic stress protocol, the susceptible and resilient groups show different VTA neuronal activity. For example, 10 days of CSDS significantly increases the in vivo spontaneous firing rates and number of bursting events in VTA DAergic neurons in susceptible mice but not in the resilient group^80^. Optogenetic induction of phasic, but not tonic, firing of the VTA DAergic neurons of mice exposed to subthreshold defeat induces a susceptible phenotype, as indicated by increased social avoidance and decreased sucrose preference^81^. Optogenetic phasic stimulation of these neurons also quickly induces a susceptible and depressive phenotype in previously resilient mice that had been subjected to CSDS^81^. In addition, NAc-projecting VTA DAergic neurons in brain slices from susceptible mice show a significantly higher firing rate than those of control and resilient mice^81^. These observations indicate that increased VTA-NAc DAergic neuronal activity with a phasic firing pattern is a key determinant of susceptibility in CSDS.

It has been proposed that HCN channels, channels that mediate *I*_h_, may be responsible for modulating the excitability of VTA DAergic neurons. Increased firing of VTA DAergic neurons after CSDS is associated with increased *I*_h_^80,82^. Intriguingly, enhancement of *I*_h_ is observed not only in susceptible mice but also in resilient mice that exhibit stable normal firing of these neurons, and *I*_h_ is even larger when potassium (K + ) channel currents are increased^80,82^. These findings suggest a possible mechanism by which resilience is homeostatically maintained by VTA DAergic neuronal activity through a compensatory upregulation of potassium channels in response to excess hyperactivity^82^.

However, unlike for other chronic stress-induced models of depression, however, contradictory results have been observed for the CUMS paradigm. Tye et al. reported that selective inhibition of VTA DAergic neurons induces multiple depression-like behaviors^83^. In this study, the authors observed that optogenetic inhibition of VTA DAergic cells induces depressive phenotypes, whereas optogenetic activation of VTA DA neurons following CUMS (for 8 to 12 weeks) reverses the depressive behaviors of the animals^83^. When they examined how CUMS influenced VTA DAergic neuronal activity, they observed that CUMS decreased the normal bursting activity of VTA DAergic neurons without changing the mean firing rate^83^. Consistent with these data, Chang and Grace reported that the CUMS-exposed rats had 50% fewer DA neuron population activity but without differences in the average firing rate or percentage of spikes in bursts as compared to control group^84^. There was a significant decrease in VTA DA neuron population activity, which represents a recruitable pool of DA neurons for burst firing and such decreases in the number of spontaneously firing DAergic neurons would affect the DA response to external stimuli^84^. On the other hand, Zhong et al. showed that the population activity, as well as the frequency of tonic and burst firing of VTA DAergic neurons, decreases as *I*_h_ is reduced in CUMS-exposed mice^85^. In association with the decrease in *I*_h_, knockdown of HCN2 in the VTA using RNA interference induces depressive-like and anxiety-like behavior, while overexpression of HCN2 in the VTA prevents CUMS-induced depressive-like behavior^85^. Thus, as observed in other experiments, the excitability of VTA DAergic neurons is critical for the regulation of CUMS-induced depressive-related behaviors.

Moreover, in this context, an important question can be asked: why have contradictory results been obtained, and why are there different patterns of VTA neuronal activity in response to different chronic stress paradigm? One explanation is the different durations of chronic stress in the different stress protocols (Table 1). Indeed, in studies on VTA neuronal activity following chronic restraint stress or CSDS, stress exposure lasts about 10 days^78,80–82^. In studies on CUMS, stress exposure occurs for 4–12 weeks^83–85^ (for in vivo recordings from the VTA, Tye et al. exposed rats to 4–6 weeks of CUMS^83^, Table 1). It appears that exposure to chronic stress for more than 4 weeks suppresses VTA neuronal activity. It is possible that the decrease in the VTA neuronal activity in CUMS-subjected animals reflects anhedonia, or unavoidable helplessness, evoked by CUMS, which involves a longer duration of repeated but variable and unpredictable stress, in these animals. Indeed, different stressors, including food/water deprivation, illumination, presentation of predator odor, overcrowding, etc., in the CUMS protocol can alter sensory and reward circuits for a long time, possibly contributing to the observed depression in VTA DA neuronal activity. This possibility raises another question: what is the physiological significance of the increase in VTA DA neuronal activity in depressed animals after CSDS compared to the suppression of VTA DA neuronal activity after CUMS? One explanation we can consider is the heterogeneity of VTA neurons, although most of the studies reported that they targeted the PBP and PN, which are the lateral subdivisions of the VTA, for in vivo recording. Stimulation of VTA neurons with differential excitability might give rise to contradictory results despite the apparent depressive behavioral phenotype. Given that VTA DAergic neurons are heterogeneous in their afferent and efferent connectivities^17,21^, it cannot be excluded that in vivo recordings from these different subdivisions might result in subtle differences. In addition, as the VTA-NAc DAergic system is well known to be activated in response to rewarding stimuli, stress-induced excitation of VTA neurons is intriguing, and these observations indicate the presence of a diverse population of DAergic neurons in the VTA. Clarifying how DAergic neurons can integrate rewarding and aversive, stressful stimuli together will be critical to understanding the stress-induced modulation of the dopaminergic VTA-NAc reward system and its impact on stress-related adaptive behavior with reward demands.

**Table 1:** Studies showing chronic stress-induced changes in the activity of VTA DAergic neurons.

Animals, stress paradigm	Change in VTA DAergic neuronal activity induced by stress	Recording method and site	Reference
Rats, chronic restraint stress 1 h/day for 10 days	Increase in DA neuron population activity, no change in firing rate	In vivo recording from the VTA: −5.3 anteroposterior (AP); −0.6 mediolateral (ML); and −6 to −9 mm dorsoventral (DV)	^78^
Mice, CSDS for 10 days	Increase in spontaneous firing rates and bursting events of VTA DA neurons in vivo in susceptible mice	In vivo recording from the VTA: −2.92 to −3.88 AP; 0.24 to 0.96 ML; and −3.5 to −4.5 DV.	^80^
Mice, CSDS for 10 days	Significant increase in the firing rate in susceptible mice compared to control and resilient mice (VTA slices)	Slice recording	^81^
Mice, CSDS for 10 days	Increase in VTA DAergic neuron firing frequency in susceptible animals	Slice recording	^82^
Rat, CUMS for 4–6 weeks (for the rest of the experiments, mice exposed to CUMS for 8–12 weeks were used)	No change in firing rate but a decrease in the proportion of spikes occurring within bursts, the duration of bursts, and the number of spikes in each burst in the VTA neurons of stressed rats	In vivo recording from the VTA: (AP), −5.8; (ML), ±0.7; and (DV), −8.2 In vivo recording from the VTA in adult male rats (4–6 weeks of CUMS)	^83^
Rat, CUMS for 4 weeks	Decreased DA neuron population activity but no differences in average firing rate or percentage of spikes in bursts	In vivo recording from the VTA: −5.3 to −5.7 mm AP; −0.6 to −1.0 mm ML; and −6.5 to −9.0 mm DV	^84^
Mice, CUMS for 5 weeks	Decreased population activity, the frequency of tonic and burst firing in VTA DAergic neurons.	In vivo recording from the VTA: −2.9 to −3.3 mm AP; 0.6 to 1.1 mm ML; and −3.9 to −4.5 mm DV	^85^

## Stress-induced changes in the DAergic system in the NAc

As discussed earlier in this paper, most NAc cells are MSNs, which can be divided into two broad categories on the basis of the type of DA receptor expressed, namely, D1R or D2R. D1R and D2R belong to the G protein-coupled receptor family and couple to the G_s_ and G_i_ signaling pathways, respectively, to play different roles in modulating reward^86,87^.

### Regulation of accumbal dopamine receptor binding and expression after chronic stress

Previous studies have reported changes in the expression or specific binding of D1R or D2R in the NAc in the brains of animals after chronic stress exposure. After daily chronic restraint stress for 1 h for 12 consecutive days, the D1R density was decreased in the NAc, as revealed by receptor autoradiography, whereas that of D2R was not altered^88^. However, in another study after repeated immobilization stress (2 h for 10 days), an increase in D2R binding in the shell of the NAc was observed^89^. Following 7–8 weeks of CUMS exposure, the levels of DA and its metabolite were elevated in the NAc but not in the striatum in animals. In addition, CUMS decreased D2R binding specifically in the NAc^69,90^. The stress-induced decrease in D2R binding was completely reversed in animals treated chronically with the antidepressant imipramine, suggesting that changes in D2R function in the NAc are responsible for CUMS-induced anhedonia and its reversal by antidepressant drugs^89^. Following a shorter period of CUMS (for 16 days), however, Ossowska et al. reported an increase in the D1R density in the limbic system^91^.

### Chronic stress-induced plasticity changes in MSNs in the NAc

A balance in the activity of D1-MSNs and D2-MSNs appears to be important for normal behavioral outputs, and it has been suggested that dysregulation resulting in an imbalance in the activity of these cell types can be implicated in the pathophysiology of depression^92^. Initial reports proposed a relatively dichotomous role for these two populations in the dorsal striatum as well as the NAc; for example, activation of the D1-MSN pathway promotes reward, while activation of the D2-MSN works promotes punishment and aversion^93,94^. However, recently, increasing data has challenged this simple dichotomy^47,95,96^, and the regulation of stress-induced changes in MSNs is rather complex.

It was previously reported that ΔfosB, a Fos family transcription factor, is induced in both dynorphin-positive (D1-MSNs) and enkephalin-positive (D2-MSNs) neurons in the NAc by chronic restraint stress^97^. Vialou et al. reported an increase in ΔFosB expression in the NAc after CSDS and showed that resilient mice exhibit the greatest induction of ΔFosB in both the core and shell of the NAc, suggesting that ΔFosB induction in the NAc is both necessary and sufficient for resilience and the response to antidepressants^98^. Through cell-type specific analysis, Lobo et al. showed that after CSDS, susceptible, depressed mice display a significant induction of ΔFosB in D2-MSNs in the NAc core, NAc shell, and dorsal striatum, while resilient mice show significant ΔFosB induction in D1-MSNs across all striatal regions^99^. Similarly, Lim et al. reported that repeated restraint stress (3–4 h per day for 7–8 days) decreases the strength of excitatory synapses on D1-MSNs but not on D2-MSNs of the NAc core, suggesting that a D1-MSN-specific change in excitatory transmission could be responsible for the induction of anhedonia^100^.

Francis et al. performed another comparative study on the differential excitatory synaptic inputs and intrinsic excitability of D1- and D2-MSNs after CSDS^101^. Excitatory synaptic inputs on MSN subtypes after CSDS were examined, and it was found that in susceptible mice that show depression-like behaviors, the frequency of excitatory synaptic input is decreased in D1-MSNs and increased in D2-MSNs^101^. Stimulation of D1-MSNs using optogenetics and pharmacogenetics resulted in a resilient behavioral phenotype, while inhibition of these MSNs induced depression-like behaviors after CSDS^101^. Khibnik et al. demonstrated that after CSDS, resilient animals display an increase in synaptic strength at large mushroom spines of D1-MSNs and a concomitant decrease in synaptic strength at those of D2-MSNs; however, in this study, susceptible mice did not exhibit a significant change in synaptic strength at D1-MSNs or D2-MSNs^102^. These observations raise the possibility that depressive behaviors can be managed by targeting D1-MSNs; however, the role of D2-MSNs in regulating depression remains questionable, despite the subtle inverse regulation of these neurons compared to D1-MSNs.

Dias et al. reported that an increase in the signaling of β-catenin, a multifunctional protein, occurs in D2-MSNs in resilient mice after 10 days of CSDS, while a decrease in β-catenin signaling is seen in susceptible animals^103^. It appears that β-catenin in D2-MSNs activates a network in the NAc that mediates resilience to chronic stress, whereas deficits in this pathway contribute to depression-related pathology^103^. These data suggest a role for D2-MSNs in controlling depressive behaviors that may include β-catenin-mediated changes in gene expression and synaptic plasticity in D2-MSNs.

In association with the chronic stress-induced changes in the excitability of MSNs, several studies have reported changes in dendritic spine structure and density, which involve various neurotrophic factors, cell adhesion molecules, and kinases^104–107^. However, these findings are contradictory in terms of the correlation between these structural changes in spines and depressive/resilient behavior, which thus remains to be elucidated (Table 2).

**Table 2:** Plasticity changes in NAc MSNs induced by chronic stress.

Animals, stress paradigm	Plasticity changes in NAc MSNs induced by chronic stress	Reference
Rats, chronic restraint stress 1 h/day for 10 days	∆FosB was induced in both dynorphin-positive (D1-MSNs) and enkephalin-positive (D2-MSNs) by stress	^97^
Mice, CSDS for 10 days	Resilient mice showed the greatest induction of ΔFosB in both the core and shell of the NAc	^98^
Mice (*Drd1-*EGFP and *Drd2-*EGFP for the MSN study), CSDS for 10 days	Depressed mice displayed a significant induction of ΔFosB in D2-MSNs in the NAc core, NAc shell, and dorsal striatum; resilient mice showed significant ΔFosB induction in D1-MSNs across all striatal regions	^99^
Mice (*Drd1*–tdTomato and *Drd2*-EGFP), chronic restraint stress 3–4 h/day for 7–8 days	The strength of excitatory synapses on D1-MSNs in the NAc core was decreased	^100^
Mice (*Drd1-*EGFP and *Drd2-*EGFP), CSDS for 10 days	The frequency of excitatory synaptic inputs was decreased in D1-MSNs and increased in D2-MSNs	^101^
Mice *(Drd2-*EGFP), CSDS for 10 days	Resilient animals displayed an increase in synaptic strength at large mushroom spines of D1-MSNs and a concomitant decrease in synaptic strength at D2-MSNs	^102^
Mice *(Drd1-*EGFP and *Drd2-*EGFP), CSDS for 10 days	β-catenin expression was upregulated in D2-MSNs in resilient mice but downregulated in susceptible animals	^103^
Mice, CSDS for 10 days	mEPSC frequency was increased, and this increase was associated with significant increases in IκB kinase expression in the NAc in susceptible (depressed) animals	^104^
Rats, CUMS for 6 weeks,	Medium spiny neurons in the NAc were hypertrophied and showed increased expression of genes encoding brain-derived neurotrophic factor and neural cell adhesion molecule in depressed animals	^105^
Mice (D1-Cre x RiboTag (D1-Cre-RT)), CSDS for 10 days	The expression of the transcription factor early growth response 3 (EGR3) was increased in the D1-MSNs of susceptible mice	^106^
Mice, CUMS for 3 weeks	Spike timing-dependent long-term potentiation (tLTP) was induced in NAc MSNs, and the level of active glycogen-synthase kinase 3β (GSK3β) was increased in depressed mice	^107^

## Conclusions

Given these diverse results obtained using different stress-induced depression models, the role of the VTA-NAc pathway in stress-induced depression in association with reward system modulation and its consequences is unclear. Although the VTA-NAc DAergic system is well known to be activated in response to rewarding stimuli, considerable evidence indicates that VTA DAergic neurons are also excited by a variety of aversive and stressful stimuli, as discussed in the present review of stress-induced changes in the VTA-NAc DAergic system. Recent findings suggest the presence of a diverse population of DAergic neurons, or at least a population that is excited by reward and inhibited by aversive stimuli (motivational value)^16,108,109^, which control approaches to rewards and the avoidance of aversive stimuli, thus providing value learning. Another population excited by both reward and aversive events in a similar manner (motivational salience) having weaker responses to neutral events^16,108,109^. These neurons are critical for orienting attention and for selective information processing for an optimal outcome. In addition, considerable evidences have shown that DAergic neurons respond to salient and arousing change in environmental conditions that are not necessarily associated with the reward value^110^. These changes include several types of sensory events, surprise, novelty, arousal, attention, and salience; therefore, these alerting signals can excite DAergic neurons^16,110^. It has been hypothesized that these value, salience, and alerting signals cooperate to coordinate and control motivated behavior^16,108,109^, which may ultimately be important not only for reward value but also for supporting specific forms of adaptive behavior to react and cope with changes in the environment^16^ (Fig. 3).

This hypothesis can provide a reasonable explanation for why both rewarding and stressful stimuli can excite the VTA-NAc DAergic system. However, the neurons and molecules and signaling pathways that are responsible for chronic stress-induced modulation of the VTA-NAc system and the resulting depression and anhedonia are unclear. In particular, the discrepancies in the role of VTA DAergic neuronal activity between CSDS-induced and CUMS-induced depression are intriguing. As mentioned above, these two chronic stress protocols have different exposure durations; in addition to lasting for a longer time, CUMS involves variable and unpredictable stressors, including food/water deprivation, light illumination, the presentation of predator odor, overcrowding, etc. It is plausible that differential excitation/inhibition among these value/salience/alerting DAergic neurons may reflect the discrepancy in VTA excitability in animals with CSDS-induced and CUMS-induced depression; however, all of these chronic stress paradigms induce maladaptive behavioral consequences, such as depression.

Together with the cytochemical and molecular heterogeneity of VTA DAergic neurons, the complex anatomical connectivity of the NAc makes it difficult to fully understand this system. With current breakthroughs in cell type- and circuit-specific genetic manipulation and neuronal imaging tools, it will likely be possible to dissect some of the specific neuronal ensembles that encode reward value or salience among DAergic neurons. In addition, we have to admit that stress-induced depression and anhedonia are actually more complicated than we had proposed, and studies seeking to identify the neuronal components and connectivity associated with behaviors related to reward value/salience of aversion in the DAergic system will certainly have further important implications for clinical investigations.
